# Supplementary material for: Has resistance to chlorhexidine increased among clinically-relevant bacteria? A systematic review of time course and subpopulation data
Source: PLoS One. 2021 Aug 19;16(8):e0256336. doi: 10.1371/journal.pone.0256336 (PMC8376095; doi:10.1371/journal.pone.0256336)
Supplement: S1 Appendix — (DOCX) [file pone.0256336.s002.docx]

Supplemental Material, Appendix S1

Statistics for analysis of minimum inhibitory concentration (MIC) data

**Problems in analysis of minimum inhibitory concentration (MIC) data**

The use of MICs to measure anti-microbial activity has been the most frequently used method for assessing antimicrobial potency for approximately 100 years. However, there are inherent statistical and analytical limitations to the use of MICs as typically applied. Nearly all antimicrobial sensitivity data have been generated using dilution series. Two significant problems are, first, MIC values almost always are observed as a series of discrete values rather than as continuous values contrary to underlying assumptions of many statistical techniques. This frequently includes multiple identical discrete values appearing many times in a data set for different strains of an organism as well as the same values frequently found in multiple reports. Seldom is an attempt made to define the inhibition values more precisely. Additionally, the intervals between measured values are not uniform. For example, MIC values of 1 and 2 µg/ml have a range four-fold larger than the range between 0.25 and 0.5 µg/ml. Analysis can be further complicated when individual MIC values are not presented, but MIC_50_ and or MIC_90_ values may be used to characterize a group of microbial strains.

MIC data are “censored”. That is, a true MIC value may lie above the stated MIC value in a serial dilution. Thus, for an MIC measured as 16 µg/ml the true MIC is probably greater than 16 µg/ml but less than the next higher dilution in the series, e.g. 32 µg/ml. Typically, no greater precision is attempted. This leads to an underestimate of the actual MIC and distorts the measurement error [1] to variable degrees depending on the dilution. Censoring by use of MICs is an historical and continuing common practice. Censoring’s most important effect is probably to desensitize some analyses because of limited data. The risk is of failure to detect differences that more closely spaced concentrations would detect, although false detection of true differences is unlikely.

Fundamental assumptions of linear regression include that the data are distributed consistent with the normal (Gaussian) distribution and that there is equal variance (homoscedasticity) among groups. Use of the logarithm of MIC values converts the distribution to log-normal, and a strong argument can be made for the use of log-normal distributions [2,3] with MIC data. Histograms in the form of probability density distributions constructed from MIC data appear to resemble normal distributions, an assumption that has been applied previously with MIC data [4–6]. Base-two logarithms were selected for the work described here for ease of calculation and ease of conversion from logarithmic form. In particular, the log_2_-transformation is most useful when doubling dilutions predominate and provides a straight-forward translation from 2-fold dilutions, e. g. 0.5, 1, 2, and 4 µg/ml, to the equivalent log_2_ values of -2, -1, 0, and 2 log_2_(MIC) units.

In most reports of antimicrobial effects precise isolation dates are rarely provided. Isolation dates are typically described as occurring over a time interval, which can vary over months or years. Regression analysis makes the assumption that the independent variable – i.e. isolation date – is precisely known. When the goal is to determine if resistance to an antimicrobial has changed with time, imprecise isolation dates introduce uncertainty regarding the time course of the appearance of resistance. To reflect the appropriate degree of precision, regression analysis must take into account isolation date uncertainty so that parameters and error associated with parameters can be determined accurately.

Although a definitive theoretical basis for the relationships among statistical distribution of data, sample size, and variance homogeneity has not been developed, Monte Carlo sampling, i.e. random sampling tests from known populations to show effects of various distribution, variance and sample size differences have been used to determine effects on robustness of statistical tests.

The effects of randomizations in isolation date and/or MIC can be determined by observing the variability of parameters derived from regression analyses carried out on a series of randomized data sets. Does the slope in linear regression vary substantially when randomization of isolation times and/or MIC values are used? Variability of the slope parameter is indicated by the error – standard deviation or standard error of the mean – observed across randomized data sets termed permutation runs. A complementary test is the use of the F-test for the linear regression model, which is a test of whether the predictor provides a better fit to data than a model that contains no predictor. The F-test in this context has been described as a measure of “the explained mean square, or mean square due to linear regression” [7].

## Generating “fixed” data sets from uncertain isolation dates and uncertain MIC values

To carry out either linear regression or non-linear least squares regression, values in the data sets were assigned fixed values. To accommodate the range of possible values that isolation dates or MIC values may assume, multiple data sets were generated using constraints on dates and MIC values specified in particular articles by study authors. Thus, specific isolation dates within a listed range stated by authors were used to restrict the range of randomized values. Similarly, when MIC values were provided as MIC_50_ or MIC_90_ values, they were restricted to a few possible integer values randomly assigned, such as 1, 2, or 4 µg/ml. For values with uncertainty in date and MIC values, values were determined independently so that isolation dates and MIC values were not correlated.

Selection of specific values for dates

The simplest example is if the dates of isolation were specified as having occurred within a specific year. Random dates within the year were generated using the RAND() function in Excel, which generates a random number over the range 0 to 1 using a uniform (rectangular) distribution. Thus, any value from 0 to 1 is equally likely to be selected. The resulting function is:

Specific date = base date + RAND(), e.g. value in range 2008-2008.9999 = 2008+RAND()

If the isolation date range specified was, for example, two years, 2008 or 2009, the resulting date randomization generating function was

Value in range 2008 to end of 2009 = 2008 +2*RAND().

Fractional years were similarly assigned as, e.g. Jan 2008 to June 2009:

Specific Value = 2008 +1.5*RAND()

Multiple data sets (permutation runs) with fixed values were generated. Linear regression was then performed on the multiple data sets to account for the effect of imprecise isolation dates.

Selection of specific MIC values

Since the use of specific dilution series for manipulation of antimicrobial concentrations is nearly universal and the series is usually based on 2-fold serial dilutions, the approach was to select specific discrete values within the range specified by authors. As a simple example, if the choice was between two possible values, such as 2 or 4 µg/ml, the following Excel 2013 command series can be used to randomize between the two choices. Two columns were used, one with the values of a random number generated using the RAND() function and a second column with the selection criterion based on the value of RAND(). The format for the command in column B is: IF(random value < 0.5, choose X, else choose Y) where X = 2 and Y = 4 in the example below.

| Row # | Column A | Column B |
| --- | --- | --- |
| 1 | =RAND() | =IF(A1<0.5, 2, 4) |
| 2 | =RAND() | =IF(A2 < 0.5, 2,4) |
| 3 | =RAND() | =IF(A3 < 0.5, 2,4) |

Note that the spaces in the command are not required. The resulting three values displayed in columns B1 – B3 will be independent of each other and each will be equally likely to be either 2 or 4. Over multiple replicate permutation runs, the values in B1 – B3 will vary randomly. If the choice was among three possible values, e.g. 2, 4 or 8, the following example illustrates the procedure.

| Row # | Column A | Column B |
| --- | --- | --- |
| 4 | =RAND() | =IF(A1<1/3, 2, (IF(A1 < 2/3, 4, 8)) |
| 5 | =RAND() | =IF(A1<1/3, 2, (IF(A1 < 2/3, 4, 8)) |
| 6 | =RAND() | =IF(A1<1/3, 2, (IF(A1 < 2/3, 4, 8)) |

This will generate random discrete values of 2, 4, or 8 in each of cells B4, B5, and B6. When both isolation date and MIC values were not listed by authors as specific values, two independent RAND() functions were used to assign date and MIC values so that dates were not correlated with MIC values.

The randomization/permutation runs were used in the limited cases where data were specified as concentration ranges, MIC_50_, and MIC_90_. The table below illustrates details for assigning MIC values.

| **Table A1. Example of Values Assigned for MIC_50_, MIC_90_ and MIC-Range Data**  The example assumes n = 10, range = 0.5 to 16 µg/ml, MIC_50_ = 2, and MIC_90_ = 8. | | |
| --- | --- | --- |
| Strain number | Value(s) Assigned | Comments |
| 1 | 0.5 | Minimum in range |
| 2 | 0.5 or 1 or 2 | Randomized based on uniform distribution; if Rand() < 1/3, value = 0.5, if RAND() < 2/3, value = 1, otherwise value = 2 |
| 3 | 0.5 or 1 or 2 | Randomized based on uniform distribution; if Rand() < 1/3, value = 0.5, if RAND() < 2/3, value = 1, otherwise value = 2 |
| 4 | 0.5 or 1 or 2 | Randomized based on uniform distribution; if Rand() < 1/3, value = 0.5, if RAND() < 2/3, value = 1, otherwise value = 2 |
| 5 | 2 | Force 50% value to MIC50 |
| 6 | 2 or 4 or 8 | Randomized based on uniform distribution; if Rand() < 1/3, value = 2, if RAND() < 2/3, value = 4, otherwise value = 8 |
| 7 | 2 or 4 or 8 | Randomized based on uniform distribution; if Rand() < 1/3, value = 2, if RAND() < 2/3, value = 4, otherwise value = 8 |
| 8 | 2 or 4 or 8 | Randomized based on uniform distribution; if Rand() < 1/3, value = 2, if RAND() < 2/3, value = 4, otherwise value = 8 |
| 9 | 8 | Set 90% value to MIC90 |
| 10 | 16 | Maximum in range |

At least 50 randomizations were carried out to generate independent randomized data sets for linear regression for pooled data of each microbial species. In nearly every case, randomization of dates for a particular subset of data is short relative to the time course being tested. That is, if isolation dates were specified by authors as occurring over one, two, or even five years, this is relatively short compared to the overall 50-70 year time interval assessed by randomization. Moreover, an explicit test of variability introduced by uncertainty in isolation dates is provided by the permutation runs and calculation of the error associated with the parameters as well as explicit tests of statistical significance of the regression, i.e. the F-test mentioned above. As an example, the F-test for the linear regression model in *Ps. aeruginosa* data had p-values ranging from approximately 0.00002 to 0.0002 for 50 permutation runs. The range of parameter values determined for the various permutation runs are given in Appendix S2.

Isolation dates used for standard strains maintained by culture collections were based on information provided by the culture collection website (e.g. <https://www.atcc.org/>). Often, specific isolation dates are available from the culture collection database, at least with regard to the year of isolation. Many such strains were isolated many years ago, e.g. in the 1940s and 1950s. This makes them particularly important to include, since strains isolated, well maintained, and well documented so long ago are rare.

As indicated in Table S2, among the 10419 MIC values used in the analyses in this report, 1521 were based on permutations and a randomization of possible MIC values, i.e. ~ 15% of the total. However, 1014 of the 1521 values were based on randomized selection from one of two consecutive MIC values, i.e. ~10% of the total. Thus, MIC values with more than two possible choices were a relatively small proportion of the total values used in analyses. The robustness of the slope parameters, of the F-test of the regression model in linear regression, and of population parameters for fits of log-normal distributions to the data indicate the limited effect of the randomizations on the estimates of linear regression and log-normal fit parameters.

| **Table A2. Uncertainty in Compiled MIC Values** | | | |
| --- | --- | --- | --- |
| **Species** | **Total MIC values**  **(Number of Reports Compiled)** | **Number of MIC Randomizations**  **(% of total n)** | **Number of Randomizations**  **with 2 MIC values**  **(% of total n)** |
| *P. aeruginosa* | 523 (23) | 86 (16.4%) | 67 (12.8%) |
| *S. aureus* | 3585 (24) | 738 (20.6%) | 537 (15.0%) |
| *K. pneumoniae* | 714 (20) | 154 (21.6%) | 9 (1.3%) |
| *E. coli* | 2179 (29) | 69 (3.2%) | 25 (1.1%) |
| *A. baumannii* | 1277 (18) | 290 (22.7%) | 236 (18.5%) |
| *S. epidermidis* | 820 (14) | 81 (9.9%) | 81 (9.9%) |
| *C. albicans* | 1045 (20) | 96 (9.2%) | 52 (5.0%) |
| *E. faecalis* | 276 (20) | 7 (2.5%) | 7 (2.5%) |

Non-linear least squares regression to determine fit of log-normal distributions to MIC data

Standards applicable to bacterial resistance to conventional clinically-relevant antibiotics have been called for by international bodies including the World Health Organization, who have worked to establish a Global Antimicrobial Resistance Surveillance System (GLASS) [8], in Europe under the European Committee on Antimicrobial Susceptibility Testing (EUCAST) [9], in the United States by the FDA [10], and by other agencies around the world. However, defining resistant and multidrug-resistant standards is not yet universal [11]. One approach is to define breakpoints distinguishing either or both of clinically efficacious antibiotic concentrations from non-efficacious ones, i.e. “susceptible” and “resistant” populations [12,13]. The concept of susceptible and resistant bacterial subpopulations is applicable to biocides, but pharmacological considerations such as maximum clinically useful concentrations as used for antibiotics do not apply. Breakpoint measurements for biocides are not well developed. Nevertheless, an approach to epidemiological cut-off values (ECOFFs) for biocide resistance was described recently [14]. The authors used the concept of log-normal distributions for bacterial populations and defined ECOFFs for four common biocides, including CHX, using MIC values for more than 3000 microbial isolates.

The analysis of susceptibility to CHX in populations of strains within microbial species relied on the use of probability density functions, although authors did not specifically name the statistical form of the probability distribution used [14]. An alternative is proposed here and has been used to analyze the population data from MIC values of individual species.

There are two fundamental ways to consider a population distribution using the parameters defining a probability distribution. The probability density function is defined for a normal distribution as:

$f\left( x \right)=\frac{1}{\sqrt{2\pi}\sigma}e^{-\frac{{(x-\mu)}^{2}}{2\sigma^{2}}}$.

The integral of this function provides the cumulative probability function:

$$F\left( x \right)= \int_{-\infty}^{x} f\left( x \right)= \int_{-\infty}^{x} \frac{1}{\sqrt{2\pi} \sigma}e^{-}\frac{{(t-\mu)}^{2}}{2\sigma^{2}} dt$$

Considering the common use of 2-fold serial dilutions for expressing antibiotic or antiseptic potency, the data readily fall onto a logarithmic scale with base two. Perhaps more importantly, a variable X is said to have a lognormal distribution if Y = ln(X) is normally distributed, which is a convention adopted commonly for showing distribution of MIC data. The use of the units “log_2_(MIC)” in this report reflects this conversion. This is a modification of the normal distribution as:

$f\left( x \right)=\frac{1}{\sqrt{2\pi}\sigma}e^{-\frac{{(ln(x)-\mu)}^{2}}{2\sigma^{2}}}$

with the corresponding cumulative probability distribution containing the x to ln(x) conversion. A theoretical basis for MIC data being distributed as log-normal rather than as normal populations – or any other probability distribution - has not been much explored, although examples of apparently log-normal distributions in biology as well as in other science disciplines have been presented [2,3]. The use of log_2_ is not required; other logarithmic scales would work, including log_10_ or ln, the natural logarithm, but log_2_ is particularly convenient with the great bulk of antibiotic and antiseptic data measured using MICs obtained from 2-fold serial dilutions.

From a practical perspective, the probability density function or frequency distribution has a shape sometimes termed the “bell-shaped curve”. The corresponding cumulative distribution function is a continuously increasing function from left to right. Either function may be used to characterize populations. However, a significant practical problem with the density function is the need to define bins. There is no fundamental theoretical basis for selecting bins. Both the number of bins and choosing bin boundaries is unclear. However, all binning results in a loss of information, so avoiding the practice may be beneficial [15]. The cumulative distribution function has no such limitation since it only requires that values be placed in increasing cumulative order to form the continuously increasing result.

For data compiled for this report, log_2_(MIC) values for a population of interest were combined and sorted from smallest to largest to begin analysis of the fit of a single or the sum of two log-normal distributions. There are a large number of repeated values corresponding to the common log_2_ intervals. There is more than one way to deal with the repeated values but the method chosen for this work was to sequentially assign consecutive numbers through the repeated values as part of the sequential ordering of the complete data set. The minimization procedure inherent in non-linear least squares regression results in forcing the best fit values through the mean of the sequential values associated with a single log_2_(MIC) value. This is demonstrated in figures showing the results of the procedure. The number of repeated values is indicated by the number of individual symbols located at the same log_2_(MIC) value on a graph. Analytical robustness is indicated by the limited range of values for parameters observed using permutation runs wherein individual values for log_2_(MIC)s were varied within the accuracy limits provided by authors for data reported as MIC_50_ and/or MIC_90_ values.

Parameter values typically varied 10% or less across all permutation runs for the fit to the data of a single log-normal distribution or for the sum of two lognormal distributions (See tables in Appendix S2). The results indicate that the ~85% of MIC values which were explicitly stated by authors in their respective reports formed a firm basis for the models proposed, with additional information obtained from the less certain values provided by the permutation runs.

The software used for non-linear least squares regression was the generalized reduced gradient (GRG) nonlinear algorithm [16] incorporated in Microsoft (R) Excel 2013. MIC values were converted to log_2_(MIC) units thereby converting to log-normal distributions when using the Microsoft (R) Excel 2013 built in functions for normal (Gaussian) distributions. The equations used for fitting to the data for a single or the sum of two log-normal distributions were, respectively:

Cumulative normal value = norm.dist(x, mean, StdDev, true) * n

Cumulative normal value = norm.dist(x, mean_1_, StdDev_1_,true) * (N_tot_ – n_2_)

+ norm.dist(x, mean_2_StdDev_2_,true)*n_2_

where x = observed log_2_(MIC) value

mean = parameter value for the respective single (mean) or first (mean_1_) or second (mean_2_) in the log-normal distribution function

StdDev = parameter value for the respective single (standard deviation) or first (standard deviation_1_) or second (standard deviation_2_) in the log-normal distribution function

N_tot_ = total number of log_2_(MIC) values for a particular species of microorganism

n_2_ = number of log_2_(MIC) values associated with the second log-normal distribution, i.e. the subpopulation with greater resistance to CHX.

In order to compare the fit of a single or the sum of two log-normal distributions to log_2_(MIC) data, both a parametric and a non-parametric procedure were used. The parametric statistic used was the conventional F-test for comparing two models with different numbers of parameters [17]. The formula used was:

$F= \frac{\frac{\left( {SS}_{1}-{SS}_{2} \right)}{\left( {df}_{1}-{df}_{2} \right)}}{\frac{{SS}_{2}}{{df}_{2}}}$,

where SS_1_ and SS_2_ are sums of squares for the model with fewer and more parameters, respectively, and df_1_ and df_2_ are degrees of freedom for the respective models. The corresponding p-values can be determined from tables in conventional statistics texts or calculated using algorithms embedded in programs, such as Microsoft (R) Excel. Results are recorded as F- values and/or p-values for the corresponding sums of squares and degrees of freedom.

The non-parametric method used was the Akaike Information Criterion (AIC), which is based on concepts in information theory [18]. The AIC formula is:

$AIC=n*ln\left( \frac{SSE}{n} \right)+2k$ ,

where SSE is the sum of squared errors, n is the sample size, and k is the number of predictors/parameters in the model. As a non-parametric technique, there is no scale for determining the degree of difference in AIC that clearly indicates that one model is superior to another. The model with the smallest AIC is considered as the model of best fit. Nevertheless, a general rule of thumb is that a difference between AICs calculated from two models with a numerical value of 2 or greater indicates that the lower AIC corresponds to the model with superior fit to the data. In the work presented herein results are summarized as the difference between AICs for two models subtracting the model with the largest number of parameters from the model with the fewest number of parameters, abbreviated as “AIC diff”. The range of AIC differences obtained over all permutation runs for a particular data set is included in summary tables.

Citations in Appendix S1

1. van de Kassteele J, van Santen-Verheuvel MG, Koedijk FDH, van Dam AP, van der Sande MAB, de Neeling AJ. New Statistical Technique for Analyzing MIC-Based Susceptibility Data. Antimicrob Agents Chemother. 2012;56: 1557. doi:10.1128/AAC.05777-11

2. Limpert E, Stahel WA. Problems with Using the Normal Distribution – and Ways to Improve Quality and Efficiency of Data Analysis. PLOS ONE. 2011;6: e21403. doi:10.1371/journal.pone.0021403

3. Limpert E, Stahel WA, Abbt M. Log-normal Distributions across the Sciences: Keys and CluesOn the charms of statistics, and how mechanical models resembling gambling machines offer a link to a handy way to characterize log-normal distributions, which can provide deeper insight into variability and probability—normal or log-normal: That is the question. BioScience. 2001;51: 341–352. doi:10.1641/0006-3568(2001)051[0341:LNDATS]2.0.CO;2

4. Annis DH, Craig BA. Statistical properties and inference of the antimicrobial MIC test. Statistics in Medicine. 2005;24: 3631–3644. doi:10.1002/sim.2207

5. Mouton JW. Breakpoints: current practice and future perspectives. International Journal of Antimicrobial Agents. 2002;19: 323–331. doi:10.1016/S0924-8579(02)00028-6

6. Turnidge J, Kahlmeter G, Kronvall G. Statistical characterisation of bacterial wild-type MIC value distributions and the determination of epidemiological cut-off values. Clinical Microbiology and Infection. 2006;12: 418–425. doi:10.1111/j.1469-0691.2006.01377.x

7. Sokal RR, Rohlf FJ. Biometry. Second Edition. San Francisco: W.H. Freeman And Company; 1981.

8. GLASS | Global antimicrobial resistance surveillance system (GLASS) report. In: WHO [Internet]. [cited 1 May 2019]. Available: http://www.who.int/glass/resources/publications/early-implementation-report/en/

9. EUCAST: Resistance mechanisms. [cited 1 May 2019]. Available: http://www.eucast.org/resistance_mechanisms/

10. Medicine C for V. The National Antimicrobial Resistance Monitoring System. In: FDA [Internet]. 26 Apr 2019 [cited 1 May 2019]. Available: /animal-veterinary/antimicrobial-resistance/national-antimicrobial-resistance-monitoring-system

11. Magiorakos A-P, Srinivasan A, Carey RB, Carmeli Y, Falagas ME, Giske CG, et al. Multidrug-resistant, extensively drug-resistant and pandrug-resistant bacteria: an international expert proposal for interim standard definitions for acquired resistance. Clinical Microbiology and Infection. 2012;18: 268–281. doi:10.1111/j.1469-0691.2011.03570.x

12. Dalhoff A, Ambrose PG, Mouton JW. A Long Journey from Minimum Inhibitory Concentration Testing to Clinically Predictive Breakpoints: Deterministic and Probabilistic Approaches in Deriving Breakpoints. Infection. 2009;37: 296–305. doi:10.1007/s15010-009-7108-9

13. Turnidge J, Paterson DL. Setting and Revising Antibacterial Susceptibility Breakpoints. Clin Microbiol Rev. 2007;20: 391. doi:10.1128/CMR.00047-06

14. Morrissey I, Oggioni MR, Knight D, Curiao T, Coque T, Kalkanci A, et al. Evaluation of epidemiological cut-off values indicates that biocide resistant subpopulations are uncommon in natural isolates of clinically-relevant microorganisms. PloS one. 2014;9: e86669–e86669. doi:10.1371/journal.pone.0086669

15. Newman M. Power laws, Pareto distributions and Zipf’s law. Contemporary Physics. 2005;46: 323–351. doi:10.1080/00107510500052444

16. Lasdon LS, Waren AD, Jain A, Ratner M. Design and Testing of a Generalized Reduced Gradient Code for Nonlinear Programming. ACM Trans Math Softw. 1978;4: 34–50. doi:10.1145/355769.355773

17. Pettersson G, Pettersson I. Statistical methods for determination of empirical rate equations for enzyme reactions. Acta Chem Scand. 1970;24: 1275–1286. doi:10.3891/acta.chem.scand.24-1275

18. Akaike H. Information Theory and an Extension of the Maximum Likelihood Principle. In: Parzen E, Tanabe K, Kitagawa G, editors. Selected Papers of Hirotugu Akaike. New York, NY: Springer New York; 1998. pp. 199–213. doi:10.1007/978-1-4612-1694-0_15
