# Supplementary material for: Has resistance to chlorhexidine increased among clinically-relevant bacteria? A systematic review of time course and subpopulation data
Source: PLoS One. 2021 Aug 19;16(8):e0256336. doi: 10.1371/journal.pone.0256336 (PMC8376095; doi:10.1371/journal.pone.0256336)
Supplement: S2 Appendix — S1 Table. Linear regression parameters, Ps. aeruginosa, all data. S1 Fig. Distribution of p-values (F-statistic) for slope parameters, Ps. aeruginosa all data. S2 Table. Log-normal parameters for Ps. aeruginosa, all dates. S3 Table. Log-normal parameters for Ps. aeruginosa, pre-1990. S4 Table. Log-normal parameters for Ps. aeruginosa, post-1990. S2 Fig. Fit of log-normal models to Ps. aeruginosa data, post-1990. S5 Table. Linear regression parameters, K. pneumoniae, all dates. S6 Table. Log-normal parameters for K. pneumoniae, all dates. S3 Fig. Fit of log-normal models to K. pneumoniae, antibiotic resistant strains. S7 Table. Log-normal parameters for K. pneumoniae, antibiotic resistant strains. S8 Table. Linear regression parameters, A. baumannii, all dates. S9 Table. Log-normal parameters for A. baumannii, all dates. S4 Fig. Time-course of log2(MIC) values for A. baumannii strains isolated from 2010–2017. S10 Table. Linear regression parameters, A. baumannii, 2010 to 2017. S11 Table. Log-normal parameters for A. baumannii strains isolated after 2010. S5 Fig. Fit of log-normal models to A. baumannii strains isolated after 2010. S12 Table. Log-normal parameters for A. baumannii multi-drug resistant strains isolated after 2010. S13 Table. Linear regression parameters, E. coli, all dates. S6 Fig. Fit of log-normal model to E. coli, all dates. S14 Table. Log-normal parameters for E. coli, all dates. S15 Table. Analysis of antibiotic-resistant E. coli strains. S7 Fig. Time-course of log2(MIC) values for E. faecalis strains, all dates. S16 Table. Linear regression parameters, E. faecalis, all dates. S17 Table. Log-normal parameters for E. faecalis, all dates. S8 Fig. Fit of log-normal model to E. faecalis, all dates. S9 Fig. Time-course of log2(MIC) values for S. epidermidis strains, all dates. S18 Table. Linear regression parameters, S. epidermidis, all dates. S10 Fig. Fit of log-normal model to S. epidermidis, all dates. S19 Table. Log-normal parameters for S. epiderm [file pone.0256336.s003.docx]

# Supplemental Material, Appendix S2: Detailed species information

## *Ps. aeruginosa*

Data were compiled from 21 reports with a total of 523 MIC values used for further evaluations. Sixteen studies each had 10 or more MIC values used for analyses.

Parameters for linear regression for time versus MICs are given in the main text. The distribution of p-values observed for 100 permutations runs is shown in Fig. S1.

| **Table S1. Linear Regression Parameters, *Ps. aeruginosa*, all data**  (n = 523; 100 permutation runs) | | | |
| --- | --- | --- | --- |
| **Parameter** | **Mean Value** | **Range** | **Std Dev of Parameter (Std Error of the mean)** |
| **Slope** | 0.019 | 0.018 to 0.021 | 0.0048 (0.0005) |
| **r^2^** | 0.031 | 0.024 to 0.036 | 0.0022 (0.0002) |
| **F** | 16.4 | 13 to 20 |  |
| **p-value of F** | 7 x 10^-5^ | 1 x 10 ^-5^ to 2 x 10 ^-4^ |  |
| **r** | 0.175 |  |  |


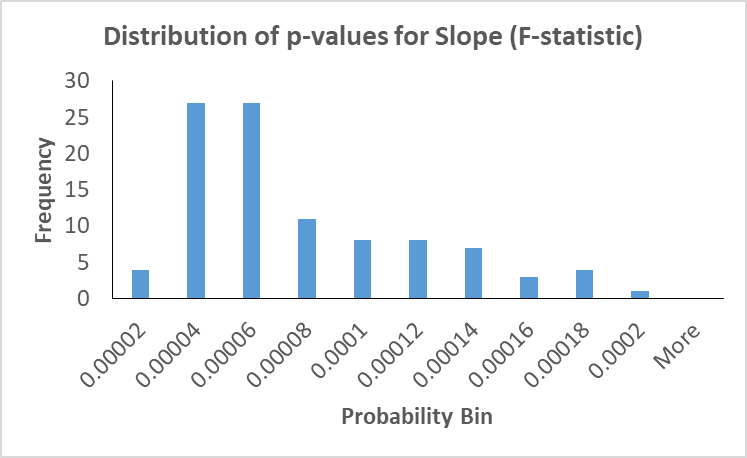


Figure S1. Distribution of p-values for F-test of slope, *Ps. aeruginosa*.

The fit to the sum of two log-normal distributions is substantially improved compared to the fit of a single log-normal distribution. Moreover, the values for the lower mean are essentially equivalent to the parameter values associated with the data from the strains isolated prior to 1990. The results are consistent with the conclusion that the post-1990 population of clinical strains contains a subpopulation with parameters equivalent to the parameters for the pre-1990 population of strains, although the total population of strains predominantly has parameters equivalent to the higher mean log_2_(MIC) population. The results are also consistent with the change from mean of 13 µg/ml in 1965 to mean of 26 µg/ml in 2015 for the strains isolated pre-1990 versus post-1990 observed in linear regression. To further test this, strains isolated after 1990 were also tested for conformance to one or the sum of two log-normal distributions. Log_2_(MIC) values compiled from strains isolated after 1990 are shown in the graph below with parameters calculated from non-linear least squares regression for two models shown in Table S1. One model used a single log-normal distribution fitted to the data and the second model used the sum of two log-normal distributions. The observed data and the best fit of both models is shown in Figure S2.

| **Table S2. Log-Normal Parameters for *Ps. aeruginosa*, all dates**  Log_2_(MIC) values (n = 523) | | | |
| --- | --- | --- | --- |
| **Parameter** | **Raw data** | **Fit of 1 log-norm** | **Fit of sum of 2 log-norms** |
| **Average Mean 1** | 4.44 | 4.30 | 3.85 |
| **Range Mean 1** | 4.42 to 4.46 | 4.28 to 4.32 | 3.83 to 3.88 |
| **Average Std Dev** | 1.63 | 1.45 | 0.69 |
| **Range Std Dev 1** | 1.61 to 1.64 | 1.43 to 1.48 | 0.63 to 0.86 |
|  |  | **Average N (Mean 2)** | 322 |
|  |  | **Range N** | 289 to 342 |
|  |  | **Average Mean 2** | 4.81 |
|  |  | **Range Mean 2** | 4.76 to 4.91 |
|  |  | **Average Std Dev 2** | 1.97 |
|  |  | **Range Std Dev 2** | 1.92 to 2.00 |
|  |  | **AIC diff range** | 162 to 255 |
|  |  | **F prob range (p-value)** | 9 x 10­^-56^ to 8 x 10 ^-36^ |

Range of parameters based on 10 permutation runs.

| **Table S3. Log-Normal Parameters for *Ps. aeruginosa*, Pre-1990**  Log_2_(MIC) values (n = 124) | | | |
| --- | --- | --- | --- |
| **Parameter** | **Raw data** | **Fit of 1 log-norm** | **Fit of sum of 2 log-norms** |
| **Average Mean 1** | 4.11 | 3.99 | Did not converge |
| **Range Mean 1** | 4.10 to 4.13 | 3.99 to 3.99 | to |
| **Average Std Dev** | 1.13 | 0.88 | Did not converge |
| **Range Std Dev 1** | 1.10 to 1.16 | 0.88 to 0.88 | to |

Range of parameters based on 10 permutation runs.

| **Table S4. Log-Normal Parameters for *Ps. aeruginosa*, Post-1990**  Log_2_(MIC) values (n = 399) | | | |
| --- | --- | --- | --- |
| **Parameter** | **Raw data** | **Fit of 1 log-norm** | **Fit of sum of 2 log-norms** |
| **Average Mean 1** | 4.55 | 4.43 | 3.84 |
| **Range Mean 1** | 4.52 to 4.57 | 4.40 to 4.46 | 3.74 to 3.95 |
| **Average Std Dev** | 1.74 | 1.65 | 0.57 |
| **Range Std Dev 1** | 1.72 to 1.76 | 1.62 to 1.70 | 0.22 to 0.83 |
|  |  | **Average N (Mean 2)** | 301 |
|  |  | **Range N** | 275 to 333 |
|  |  | **Average Mean 2** | 4.77 |
|  |  | **Range Mean 2** | 4.60 to 4.91 |
|  |  | **Average Std Dev 2** | 1.95 |
|  |  | **Range Std Dev 2** | 1.90 to 2.00 |
|  |  | **AIC diff range** | 128 to 165 |
|  |  | **F prob range (p-value)** | 1 x 10^-36^ to 1 x 10^-28^ |

Range of parameters based on 10 permutation runs.


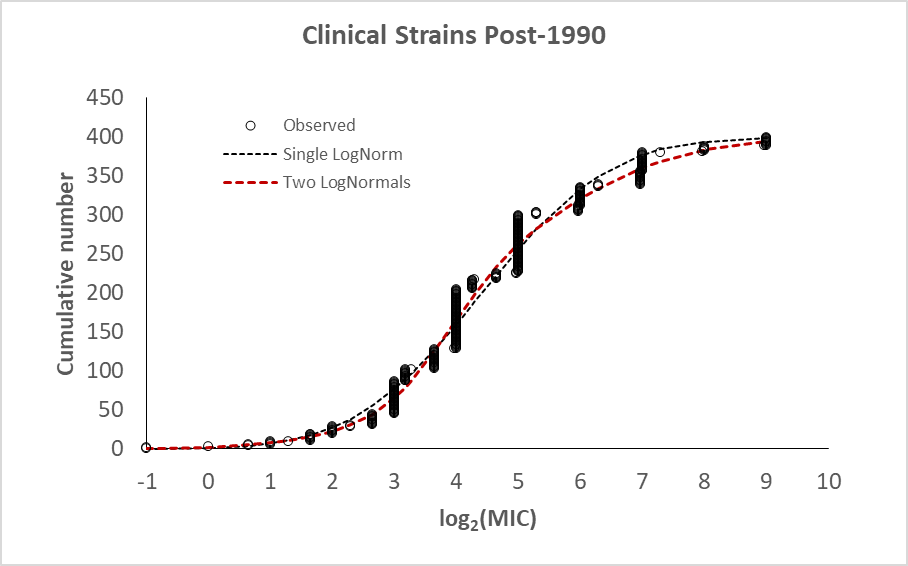


**Figure S2. Log_2_(MIC) values agreement with models with a single and the sum of two log-normal distributions.** Non-linear least squares regression was used to fit models for a single log-normal (black dashed line) and the sum of two log-normal distributions (red dashed line) to CHX log_2_(MIC)s for Ps. aeruginosa strains isolated after 1990 (n = 523) (open circles). The improved agreement with the model of the sum of two log-normal distributions was shown by the decrease in AIC ranging from 162 to 255 and p-values for the F-test comparing the two models ranging from 10^-56^ to 10^-36^.

The parameters for the first of the sum of two log-normal distributions closely agree with the values determined from the fit to the data for strains isolated before 1990. The proportion of strains with the lower MIC is approximately 30% of the strains isolated, i.e. 123 out of 399. Approximately 60% of the strains isolated after 1990 showed a slight increase in MICs for CHX, i.e. an increase from log_2_(MIC) ≈ 3.8 to log_2_(MIC) ≈ 4.8. The values are consistent with the estimates for changes in MIC for *Ps. aeruginosa* as shown using linear regression (Figure 1).

Conclusions based on both the analysis of the log-normal statistical distributions of the isolated clinical strains and from the linear regression time-course are consistent in indicating the presence of two populations of *Ps. aeruginosa* for strains isolated after 1990. The magnitude of the increase in MICs for CHX are approximately 2-fold, i.e. from a mean of ~14 µg/ml to ~28 µg/ml.

## *K. pneumoniae*

Data were compiled from 21 reports with a total of 714 MIC values used for further evaluations. Thirteen studies each had 10 or more MIC values.

| **Table S5. Linear Regression Parameters, *K. pneumoniae*** (n = 714) | | | |
| --- | --- | --- | --- |
| **Parameter** | **Mean Value** | **Range** | **Std Dev of Parameter (Std Error of the mean)** |
| **Slope** | 0.030 | 0.025 to 0.031 | 0.0010 (0.00015) |
| **r^2^** | 0.048 | 0.034 to 0.053 | 0.0034 (0.00048) |
| **F** | 35.7 | 24.4 to 39.5 | - |
| **p-value of F** | 2 x 10^-15^ | 5 x 10^-11^ to 1 x 10^-14^ | - |
| **r** | 0.220 | - | - |

Analysis of the fit of one or the sum of two log-normal distributions to the data showed significant evidence of more than a single population of strains present in the dataset of log_2_(MIC) values compiled across all dates (Table S6).

| **Table S6. Log-Normal Parameters for *K. pneumoniae*, all dates**  Log_2_(MIC) values (n = 714) | | | |
| --- | --- | --- | --- |
| **Parameter** | **Raw data** | **Fit of 1 log-normal** | **Fit of sum of 2 log-norms** |
| **Average Mean 1** | 4.88 | 4.88 | 3.90 |
| **Range Mean 1** | 4.83 to 4.93 | 4.82 to 4.93 | 3.75 to 4.06 |
| **Average Std Dev** | 2.27 | 1.98 | 0.54 |
| **Range Std Dev 1** | 2.22 to 2.32 | 1.93 to 2.02 | 0.49 to 0.60 |
|  |  | **Average N, (Mean 2)** | 654 |
|  |  | **Range N** | 632 to 676 |
|  |  | **Average Mean 2** | 5.02 |
|  |  | **Range Mean 2** | 4.97 to 5.09 |
|  |  | **Average Std Dev 2** | 2.05 |
|  |  | **Range Std Dev 2** | 2.01 to 2.10 |
|  |  | **AIC diff range** | 11 to 68 |
|  |  | **F prob range (p-value)** | 1.2 x 10^-15^ to 1.1 x 10^-3^ |

Range of parameters based on 80 permutation runs.

The quality of fits and differences between the two models are shown in the graph below and are indicated by the parameters Akaike Information Criterion (AIC) and the F-probability calculated using the F-test comparing models in the table above. The mean values of 3.90 and 5.02 log_2_(MIC) units indicated a subpopulation with mean susceptibility of 15 µg/ml and a second subpopulation making up approximately 80% of the total strains tested with mean susceptibility of 32 µg/ml, a 2.1-fold difference.

The log_2_(MIC)s for strains listed as antibiotic resistant or multi-drug resistant contain a population of 205 strains, which were analyzed separately to determine population parameters for this subpopulation of strains.


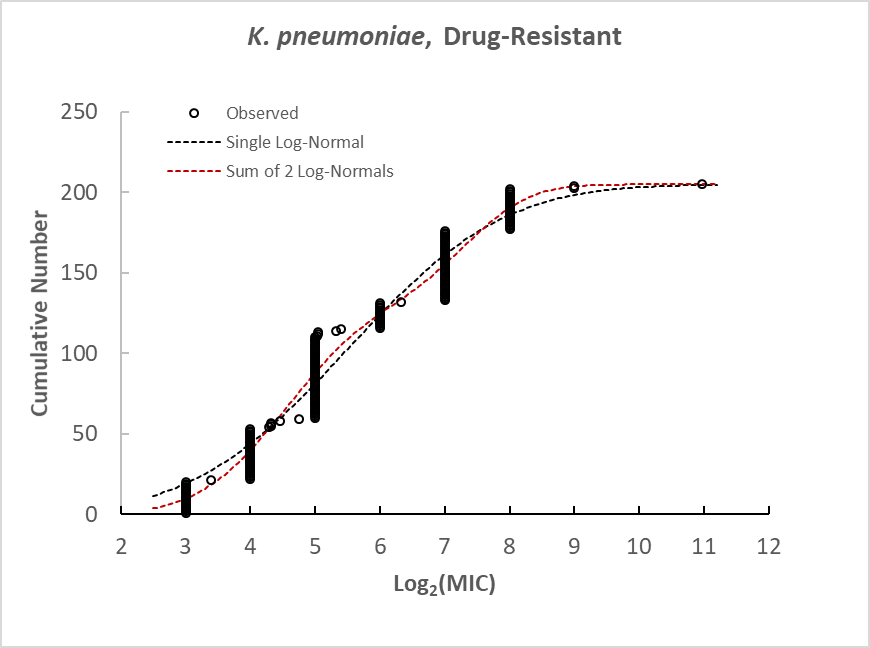


Figure S3. Fit of log-normal distributions to log_2_(MIC) values of antibiotic resistant *K. pneumoniae*.

| **Table S7. Log-Normal Parameters for Antibiotic Resistant *K. pneumoniae***  Log_2_(MIC) values (n = 205) | | | |
| --- | --- | --- | --- |
| **Parameter** | **Raw data** | **Fit of 1 log-normal** | **Fit of sum of 2 log-norms** |
| **Average Mean 1** | 5.56 | 5.50 | 4.56 |
| **Range Mean 1** | 5.51 to 5.59 | 5.44 to 5.52 | 4.36 to 4.65 |
| **Average Std Dev** | 1.62 | 1.89 | 1.07 |
| **Range Std Dev 1** | 1.60 to 1.65 | 1.85 to 1.97 | 1.01 to 1.11 |
|  |  | **Average N (Mean 2)** | 71 |
|  |  | **Range N** | 67 to 86 |
|  |  | **Average Mean 2** | 7.36 |
|  |  | **Range Mean 2** | 7.21 to 7.44 |
|  |  | **Average Std Dev 2** | 0.77 |
|  |  | **Range Std Dev 2** | 0.75 to 0.82 |
|  |  | **AIC diff range** | 80 to 187 |
|  |  | **F prob range (p-value)** | 1.1 x 10^-40^ to 6.2 x 10^-18^ |

Range of parameters based on 10 permutation runs.

The population parameters calculated using non-linear least squares regression were highly consistent across permutation runs, and both the F-test and the AIC strongly indicate that the fit of the sum of two log-normal distributions is considerably better than the fit of a single log-normal distribution. The more resistant subpopulation indicated that the population mean log_2_(MIC) = 7.4 (MIC = 55 µg/ml) and population standard deviation log_2_(MIC) = 0.8. The subpopulation corresponds to approximately 134 (65%) of the 205 strains used in the analysis. The other strains making up approximately 35% of the population had mean and standard deviation parameters consistent with parameters observed for the more resistant subpopulation in the total population (Table S6). For the data from the antibiotic-resistant strains, the fit of two log-normal distributions to the data is markedly improved over the fit of a single log-normal population with AIC reduced on average by 160, and p-values of the F-test comparing models ranging from approximately 10^-40^ to 10^-18^ (Fig. S3, Table S7).

The analysis of the known antibiotic-resistant strains separately indicated two subpopulations with regard to susceptibility to CHX, one with mean = 4.56 log_2_(MIC) units (~24 µg/ml) and a second sub-population with mean = 7.36 log_2_(MIC) units (~164 µg/ml). The corresponding standard deviations for the two subpopulations were 1.07 and 0.77 log_2_(MIC) units. If the two subpopulations are merged into a single log-normal distribution and the relative 65:35 proportion is taken into account, the combination has a mean of 5.5 and standard deviation of 1.65 log_2_(MIC) units, values reasonably consistent with the subpopulation defined in non-linear regression analysis for the second and more resistant subpopulation in the complete data set of *K. pneumoniae* MIC values (n = 712, mean = 5.3 and standard deviation = 2.0 log_2_(MIC) units). However, attempting to fit the sum of three log-normal distributions to the full (n = 712) data set did not converge on consistent values. Thus, the technique did not appear to be sensitive enough to deconvolute three separate log-normal distributions from the complete data set (n = 714) when the most resistant subpopulation was only 10% of the compiled data. Only when the antibiotic-resistant strains were analyzed separately was the third subpopulation detected.

## *A. baumannii*

Data were compiled from 18 reports with a total of 1277 MIC values used for further evaluations of *A. baumannii*. Fourteen reports each reported 10 or more MIC values. The compiled results are shown in Figure 6 and Figure 7 of the main text.

The summary parameters for linear regression for 50 permutations runs of 1277 MIC values compiled for *A. baumannii* are shown in Table S8 below. Robustness of the analysis is shown by the small variability noted for the 50 permutation runs and by the very small p-values associated with the F-test of regression. The slope is consistent with a change in log_2_(MIC) of 0.109 * 20 over a 20 year span = 2.18, which indicated a change of 4.5-fold over 20 years. Within the entire range of values for the 50 permutation runs, the change in log_2_(MIC) from a minimum of 2.12 to 2.24 (4.3 to 4.7-fold).

| **Table S8. Linear Regression Parameters *A. baumannii*, all dates** | | | |
| --- | --- | --- | --- |
| **Parameter** | **Mean Value** | **Range** | **Std Dev of Parameter (std err of the mean)** |
| **Slope** | 0.11 | 0.11 to 0.11 | 0.0011 (0.00016) |
| **r^2^** | 0.19 | 0.18 to 0.21 | 0.0050 (0.0007) |
| **F** | 307 | 282 to 332 |  |
| **p-value of F** | 2 x 10^-109^ | 3 x 10^-102^ to 6 x 10^-117^ |  |
| **r** | 0.440 |  |  |

Range of parameters based on 50 permutation runs.

The parameters for the fits of a single or the sum of two log-normal distributions to the complete data set are given in Table S9 below.

| **Table S9. Log-Normal Parameters for *A. baumannii*, all dates**  Log_2_(MIC) values (n = 1277) | | | |
| --- | --- | --- | --- |
| **Parameter** | **Raw data** | **Fit of 1 log-norm** | **Fit of sum of 2 log-norms** |
| **Average Mean 1** | 4.51 | 4.57 | 2.83 |
| **Range Mean 1** | 4.48 to 4.54 | 4.530to 4.60 | 2.78 to 2.87 |
| **Average Std Dev** | 1.49 | 1.64 | 0.78 |
| **Range Std Dev 1** | 1.48 to 1.51 | 1.62 to 1.67 | 0.74 to 0.82 |
|  |  | **Average N (Mean 2)** | 880 |
|  |  | **Range N** | 855 to 908 |
|  |  | **Average Mean 2** | 5.3 |
|  |  | **Range Mean 2** | 5.229 to 5.330 |
|  |  | **Average Std Dev 2** | 1.05 |
|  |  | **Range Std Dev 2** | 1.03 to 1.08 |
|  |  | **AIC diff range** | 529 to 646 |
|  |  | **F prob range (p-value)** | 1 x 10^-144^ to 9 x 10^-169^ |

Range of parameters based on 10 permutation runs.

The slope parameter for strains isolated in 2010 or later is nearly the same as the slope parameter of the full *A. baumannii* data set indicating that the change in resistance to CHX is detectable even in the abbreviated time span from 2010 to 2017. The correlation coefficient is consistent with F-tests for the linear regression model with very low p-values over a relatively narrow range.


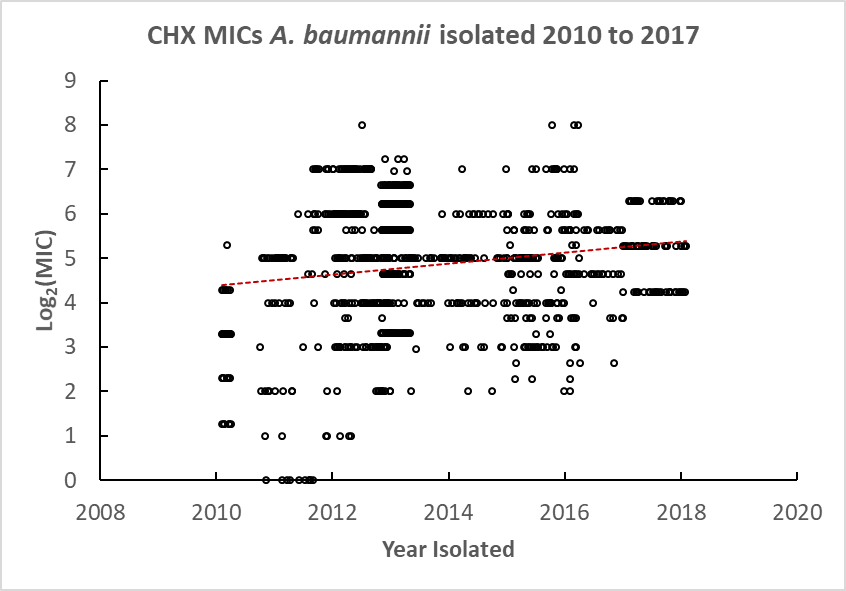


Figure S4. Time course of A. baumannii CHX log_2_(MIC)s for strains isolated from 2010 to 2017.

| **Table S10. Linear Regression Parameters *A. baumannii*, 2010 to 2017** | | | |
| --- | --- | --- | --- |
| **Parameter** | **Mean Value** | **Range** | **Std Dev of Parameter (std err of the mean)** |
| **Slope** | 0.11 | 0.095 to 0.13 | 0.0088 (0.0012) |
| **r^2^** | 0.024 | 0.0.018 to 0.032 | 0.0035 (0.0005) |
| **F** | 24.4 | 18 to 32 |  |
| **p-value of F** | 4.4 x 10^-11^ | 1.4 x 10^-8^ to 2.9 x 10^-14^ |  |
| **r** | 0.16 |  |  |

Range of parameters based on 20 permutation runs.

| **Table S11. Log-Normal Parameters for *A. baumannii*, isolation after 2010**  Log_2_(MIC) values (n = 983) | | | |
| --- | --- | --- | --- |
| **Parameter** | **Raw data** | **Fit of 1 log-norm** | **Fit of sum of 2 log-norms** |
| **Average Mean 1** | 4.87 | 4.97 | 3.90 |
| **Range Mean 1** | 4.85 to 4.90 | 4.96 to 4.99 | 3.53 to 4.10 |
| **Average Std Dev** | 1.34 | 1.42 | 1.19 |
| **Range Std Dev 1** | 1.35 to 1.42 | 1.38 to 1.44 | 0.956to 1.30 |
|  |  | **Average N (Mean 2)** | 516 |
|  |  | **Range N 2** | 452 to 634 |
|  |  | **Average Mean 2** | 5.77 |
|  |  | **Range Mean 2** | 5.66 to 5.85 |
|  |  | **Average Std Dev 2** | 0.85 |
|  |  | **Range Std Dev 2** | 0.79 to 0.90 |
|  |  | **AIC diff range** | 202 to 340 |
|  |  | **p-value for F-test** | 9 x 10^-145^ to 9 x 10^-169^ |

Range of parameters based on 10 permutation runs.


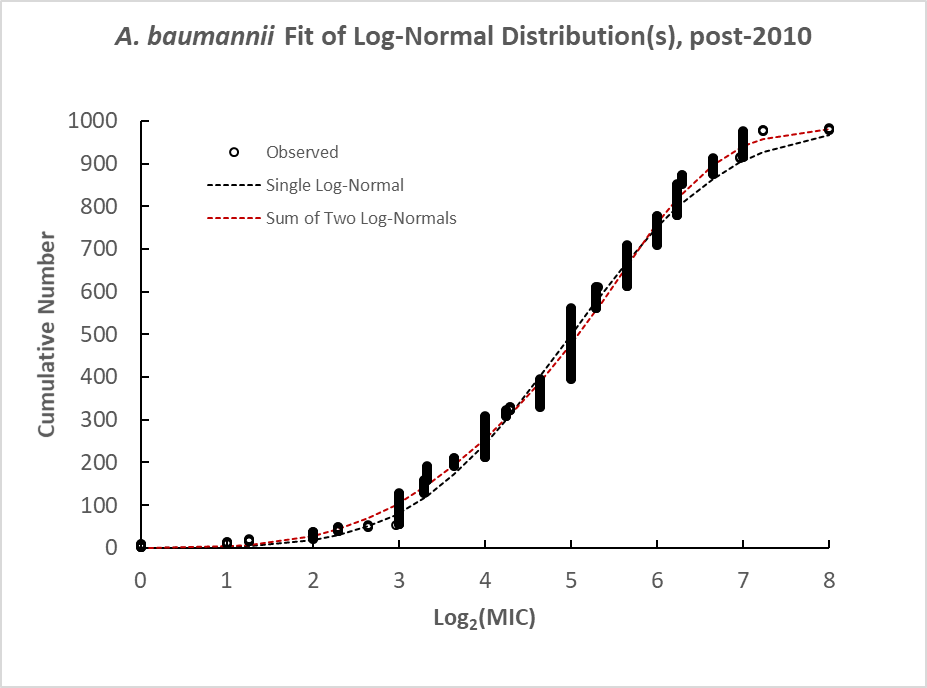


Figure S5. Fit of log-normal distributions to A. baumannii strains isolated in 2010 or later.

| **Table S12. Log-Normal Parameters for *A. baumannii*, MDR after 2010**  Log_2_(MIC) values (n = 681) | | | |
| --- | --- | --- | --- |
| **Parameter** | **Raw data** | **Fit of 1 log-norm** | **Fit of sum of 2 log-norms** |
| **Average Mean 1** | 4.95 | 5.01 | 4.36 |
| **Range Mean 1** | 4.93 to 4.97 | 4.98 to 5.04 | 4.22 to 4.54 |
| **Average Std Dev** | 1.34 | 1.44 | 1.29 |
| **Range Std Dev 1** | 1.330to 1.36 | 1.43 to 1.46 | 1.26 to 1.34 |
|  |  | **Average N (Mean 2)** | 238 |
|  |  | **Range N 2** | 167 to 282 |
|  |  | **Average Mean 2** | 6.04 |
|  |  | **Range Mean 2** | 5.939 to 6.189 |
|  |  | **Average Std Dev 2** | 0.78 |
|  |  | **Range Std Dev 2** | 0.64 to 0.86 |
|  |  | **AIC diff range** | 118 to 171 |
|  |  | **p-value for F-test** | 2 x 10^-26^ to 9 x 10^-38^ |

Range of parameters based on 10 permutation runs.

The fit to the sum of two log-normal distributions was substantially better than the fit of a single log-normal to the MIC values from the strains designated as MDR. The population parameters of the two subpopulations were mean = 4.4 (21 µg/ml) with a standard deviation parameter of 1.3 log_2_(MIC) units for the more CHX sensitive subpopulation and mean = 6.0 (64 µg/ml) and standard deviation = 0.8 log_2_(MIC) units for the more CHX-resistant subpopulation. The more resistant subpopulation appeared to constitute approximately 34.9% of the total strains designated as MDR. The results indicate that there is more than one subpopulation of *A. baumannii* strains with respect to resistance to CHX among MDR strains. The difference in CHX resistance was approximately 4-fold for the two subpopulations.

## *E. coli*

Data were compiled from 29 reports with a total of 2179 MIC values used for evaluations of *E. coli*. Thirteen reports each had 10 or more MIC values.

| **Table S13. Linear Regression Parameters, *E. coli*** | | | |
| --- | --- | --- | --- |
| **Parameter** | **Mean Value** | **Range** | **Std Dev of Parameter (std err of the mean)** |
| **Slope** | -0.035 | - 0.036 to - 0.035 | 0.0003 (0.00004) |
| **r^2^** | 0.0894 | 0.086 to 0.093 | 0.0017 (0.0002) |
| **F** | 217 | 209 to 226 |  |
| **p-value of F** | 5 x 10^-87^ | 6 x 10^-84^ to 3 x 10^-90^ |  |
| **r** | 0.299 |  |  |

Range of parameters based on 50 permutation runs.


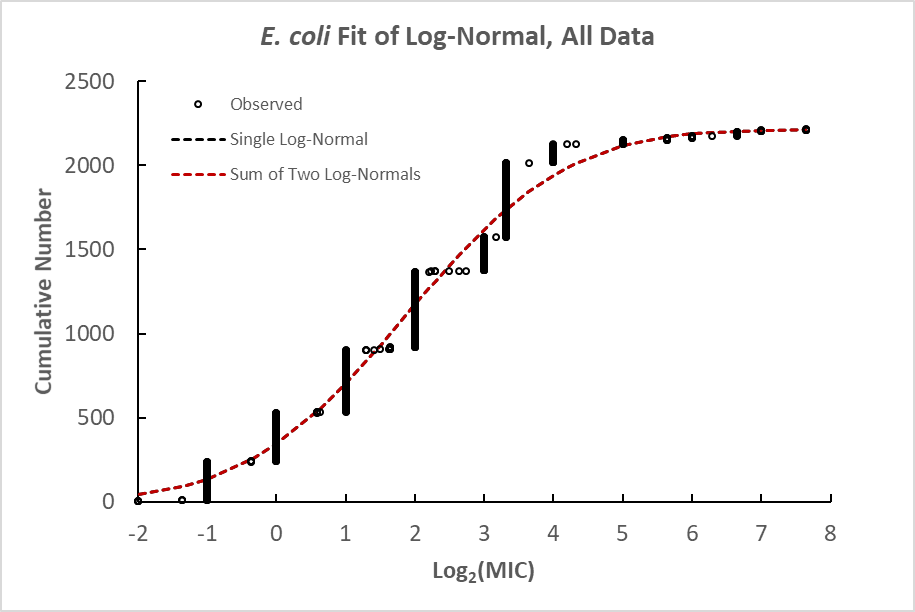


Figure S6. Fit of log-normal distribution to CHX log_2_(MIC) results for *E. coli* strains isolated for all dates.

| **Table S14. Log-Normal Parameters for *E. coli*, Pooled Data**  Log_2_(MIC) values (n = 2179) | | | |
| --- | --- | --- | --- |
| **Parameter** | **Raw data** | **Fit of 1 log-normal** | **Fit of sum of 2 log-normals** |
| **Average Mean 1** | 1.86 | 1.87 | Did not converge |
| **Range Mean 1** | 1.86 to 1.87 | 1.87 to 1.88 | to |
| **Average Std Dev** | 1.73 | 1.84 | Did not converge |
| **Range Std Dev 1** | 1.73 to 1.73 | 1.83 to 1.84 | to |

Range of parameters based on 10 permutation runs.

| **Table S15. Analysis of Antibiotic-Resistant *E. coli* Strains**  Log_2_(MIC) values (Range) | | |
| --- | --- | --- |
|  | **Resistant** | **Sensitive or Not Specified** |
| **Mean** | 4.14  (4.00 to 4.25) | 1.81  (1.81 to 1.82) |
| **Std Dev** | 1.54  (1.46 to 1.65) | 1.69  (1.69 to 1.70) |
| **n** | 45 | 2134 |
| **p-value (Welch’s t-test)** | 3.71 x 10^-13^  (1.53 x 10^-14^ to 2.01 x 10^-11^) |  |

Range of parameters based on 10 permutation runs.

## *E. faecalis*

Data were compiled from 16 reports with a total of 276 values used for further evaluations of *E. faecalis*. Seven reports each had 10 or more MIC values.


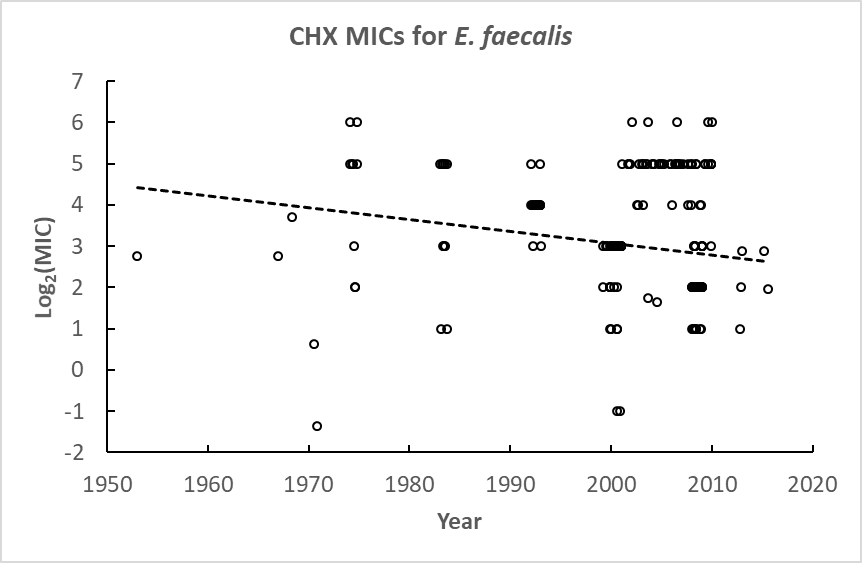


Figure S7. Time course of CHX susceptibility for strains of *E. faecalis*.

| **Table S16. Linear Regression Parameters, *E. faecalis*** | | | |
| --- | --- | --- | --- |
| **Parameter** | **Mean Value** | **Range** | **Std Dev of Parameter**  **(Standard error of the mean)** |
| **Slope** | -0.030 | -0.034 to -0.026 | 0.0020 (0.00004) |
| **r^2^** | 0.045 | 0.035 to 0.058 | 0.0057 (0.0001) |
| **F** | 12.8 | 9.9 to 17.0 | 1.71 |
| **p-value of F** | 4.4 x 10^-6^ | 7 x 10^-5^ to 10^-7^ |  |
| **r** | 0.211 |  |  |

Range of parameters based on 10 permutation runs.

| **Table S17. Log-Normal Parameters for *E. faecalis*, Pooled Data**  Log_2_(MIC) values (n = 276) | | | |
| --- | --- | --- | --- |
| **Parameter** | **Raw data** | **Fit of 1 log-norm** | **Fit of sum of 2 log-norms** |
| **Average Mean 1** | 3.0 | 2.89 | 2.33 |
| **Range Mean 1** | 3.0 to 3.1 | 2.89 to 2.90 | 2.31 to 2.35 |
| **Average Std Dev** | 1.39 | 1.50 | 0.88 |
| **Range Std Dev 1** | 1.38 to 1.39 | 1.50 to 1.51 | 0.86 to 0.90 |
|  |  | **Average n_2_** | 73 |
|  |  | **Range n_2_** | 71 to 75 |
|  |  | **Average Mean 2** | 4.93 |
|  |  | **Range Mean 2** | 4.90 to 4.95 |
|  |  | **Average Std Dev 2** | 0.68 |
|  |  | **Range Std Dev 2** | 0.65 to 0.70 |
|  |  | **AIC diff range** | 45.8 to 46.1 |
|  |  | **F prob range (p-value)** | 5 x 10^-11^ to 6 x 10^-11^ |

Range of parameters based on 10 permutation runs.


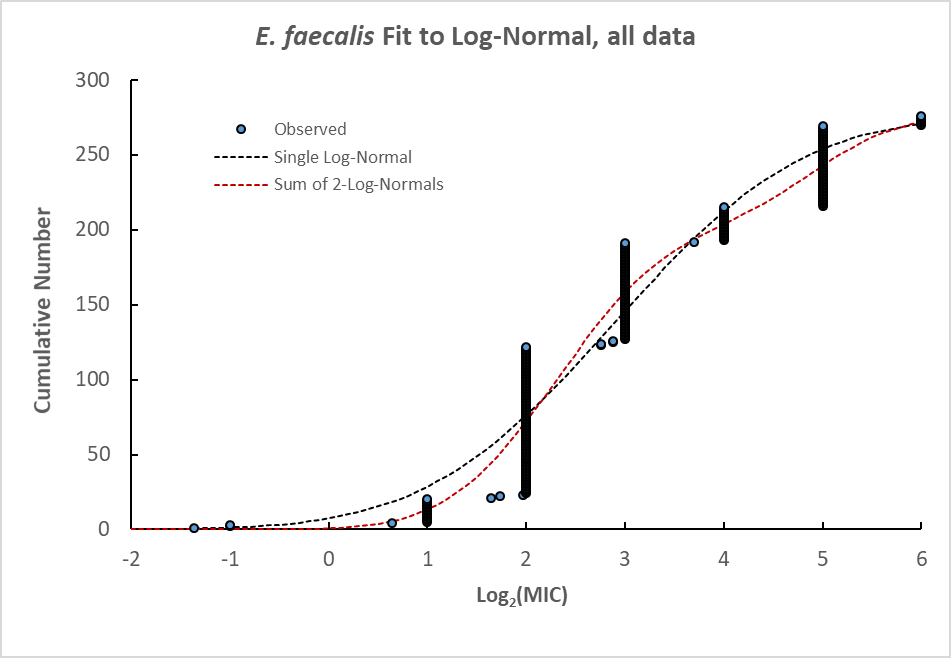


Figure S8. Fit of one or the sum of two log-normal distributions to data for strains from all isolation dates. See Table S16 for subpopulation parameters and analysis of fits.

## *S. epidermidis*

Data were compiled from 14 reports with a total of 820 values used for further evaluations of *S. epidermidis*. Six reports each had 10 or more values included in further evaluations.

**
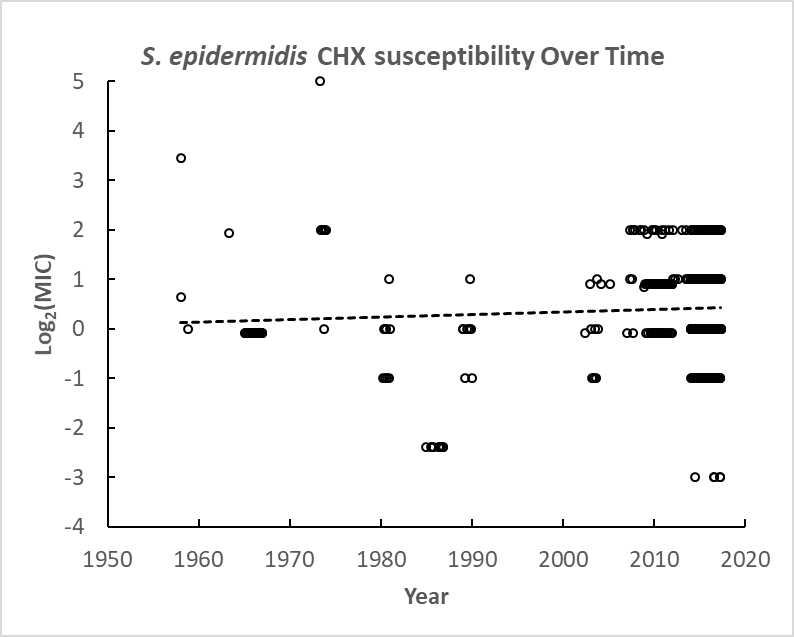
**

Figure S9. CHX susceptibility of S. epidermidis strains isolated over approximately 60 years.

| **Table S18. Linear Regression Parameters, *S. epidermidis*** (n = 820) | | | |
| --- | --- | --- | --- |
| **Parameter** | **Mean Value** | **Range** | **Std Dev of Parameter (Standard error of the mean)** |
| **Slope** | 0.0051 | 0.044 to 0.0058 | 0.00027 (0.00004) |
| **r^2^** | 0.0048 | 0.0036 to 0.0060 | 0.0005 (0.00007) |
| **F** | 3.93 | 2.97 to 4.91 | 0.41 |
| **p-value (F)** | 0.049 | 0.027 to 0.085 |  |
| r | 0.069 |  |  |

Range of parameters based on 50 permutation runs.


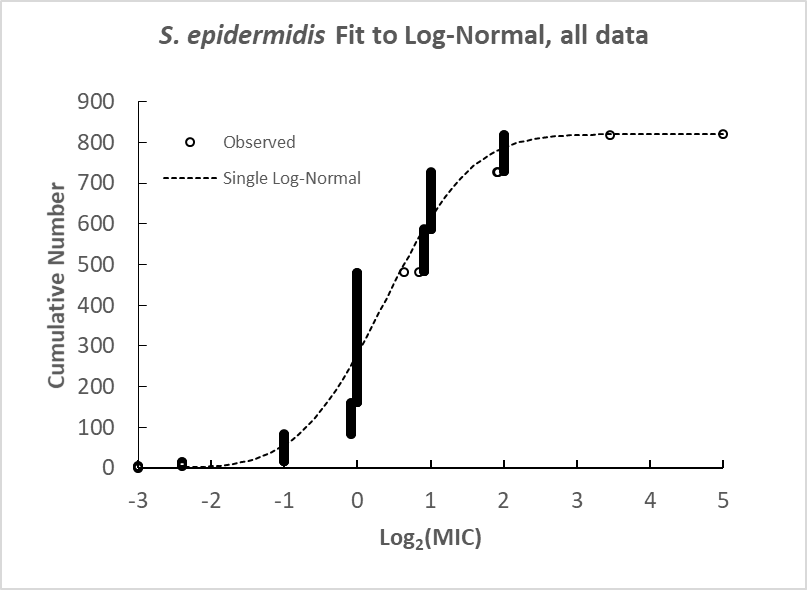


Figure S10. Fit of CHX susceptibility log_2_(MIC) values to a log-normal distribution.

| **Table S19. Log-Normal Parameters for *S. epidermidis*, Pooled Data**  Log_2_(MIC) values (n = 820) | | | |
| --- | --- | --- | --- |
| **Parameter** | **Raw data** | **Fit of 1 log-norm** | **Fit of sum of 2 log-norms** |
| **Average Mean 1** | 0.39 | 0.38 | Did not converge |
| **Range Mean 1** | 0.38 to 0.40 | 0.36 to 0.39 | to |
| **Average Std Dev** | 0.91 | 0.93 |  |
| **Range Std Dev 1** | 0.91 to 0.92 | 0.92 to 0.93 |  |

Range of parameters based on 10 permutation runs.

In the largest study included in the compilation for this report with n = 557 *S. epidermidis* strains, the authors characterized a number of strains for the presence of *qacA*, *qacB*, *smr* and various combinations of the typical antibiotic resistance factors (ref 3-6). Results were reported only as ranges of MICs and MIC_50_ and MIC_90_ values. The authors pooled antibiotic resistance factor analysis over several coagulase negative Staphylococcal species. Although *S. epidermidis* was the predominant coagulase negative species isolated (53%), *S. hominis* constituted approximately 25% of the strains isolated and analyzed for antibiotic-resistance factors and approximately 6% were *S. capitis*. The results for CHX susceptibility and the presence of antibiotic-resistance factors for *S. epidermidis* alone were not presented separately from the other coagulase-negative species. Thus, analysis of which *S. epidermidis* strains contained antibiotic-resistance factors and the CHX susceptibility of strains with or without resistance factors was not clear.

## *S. aureus*

Data were compiled from 32 reports with a total of 3585 values used for further evaluations of *S. aureus*. Twenty-eight reports each had 10 or more MIC values. The number of CHX MIC values listed as from MRSA strains was 3069, and 516 MIC values were listed as from MSSA strains.

| **Table S20. Linear Regression Parameters, *S. aureus*, MSSA** (n = 516) | | | |
| --- | --- | --- | --- |
| **Parameter** | **Mean Value** | **Range** | **Std Dev of Parameter (Std error of the mean)** |
| **Slope** | -0.0320 | -0.0292 to -0.0342 | 0.0013 (0.0002) |
| **r^2^** | 0.0865 | 0.073 to 0.101 | 0.0068 (0.0010) |
| **F** | 48.7 | 40.3 to 57.7 |  |
| **p-value of F** | 4 x 10 ^-20^ | 5 x 10 ^-17^ to 2 x 10  ^-23^ |  |
| **r** | 0.294 |  |  |

Range of parameters based on 50 permutation runs.

| **Table S21. Linear Regression Parameters, *S. aureus*, MRSA** (n = 3069) | | | |
| --- | --- | --- | --- |
| **Parameter** | **Mean Value** | **Range** | **Std Dev of Parameter (Std error of the mean)** |
| **Slope** | -0.0356 | -0.0307 to -0.0397 | 0.0021 (0.0003) |
| **r^2^** | 0.0310 | 0.0239 to 0.0369 | 0.0030 (0.0004) |
| **F** | 98.1 | 75.0 to 117 |  |
| **p-value of F** | 5 x 10 ^-42^ | 2 x 10 ^-32^ to 1 x 10 ^-49^ |  |
| **r** | 0.176 |  |  |

Range of parameters based on 50 permutation runs.

| **Table S22. Log-Normal Parameters for *S. aureus*, MRSA**  Log_2_(MIC) values (n = 3069) | | | |
| --- | --- | --- | --- |
| **Parameter** | **Raw data** | **Fit of 1 log-norm** | **Fit of sum of 2 log-norms** |
| **Average Mean 1** | 0.95 | 0.93 | Did not converge |
| **Range Mean 1** | 0.94 to 0.96 | 0.92 to 0.0.93 | to |
| **Average Std Dev** | 1.21 | 1.18 | Did not converge |
| **Range Std Dev 1** | 1.20 to 1.23 | 1.180 to 1.190 | to |

Range of parameters based on 10 permutation runs.

| **Table S23. Log-Normal Parameters for *S. aureus*, MSSA**  Log_2_(MIC) values (n = 516) | | | |
| --- | --- | --- | --- |
| **Parameter** | **Raw data** | **Fit of 1 log-norm** | **Fit of sum of 2 log-norms** |
| **Average Mean 1** | 0.35 | 0.23 | Did not converge |
| **Range Mean 1** | 0.31 to 0.38 | 0.20 to 0.26 | to |
| **Average Std Dev** | 1.51 | 1.33 | Did not converge |
| **Range Std Dev 1** | 1.46 to 1.55 | 1.31 to 1.35 |  |

Range of parameters based on 10 permutation runs.


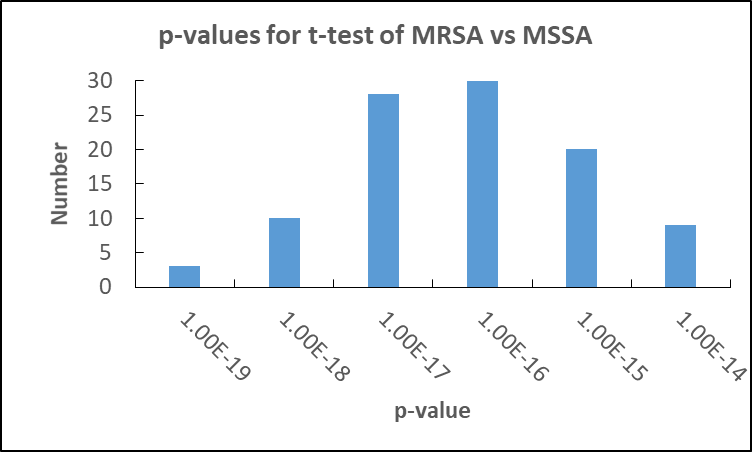


Figure S11. Distribution of p-values for t-tests comparing MRSA and MSSA log_2_(MIC) values obtained using 100 permutation runs.

## *C. albicans*

Data were compiled from 20 reports with a total of 1045 values used for further evaluations of *C. albicans*. Twelve reports each had 10 or more values.

| **Table S24. Linear Regression Parameters, *C. albicans*** | | | |
| --- | --- | --- | --- |
| **Parameter** | **Mean Value** | **Range** | **Std Dev of Parameter**  (Standard error of the mean) |
| **Slope** | -0.060 | - 0.062 to - 0.059 | 0.0006 (0.00009) |
| **r^2^** | 0.102 | 0.097 to 0.108 | 0.0023 (0.0003) |
| **F** | 119 | 112 to 126 |  |
| **p-value of F** | 1 x 10^-47^ | 7 x 10^-45^ to 8 x 10^-50^ |  |
| **r** | 0.320 |  |  |

Range of parameters based on 50 permutation runs.


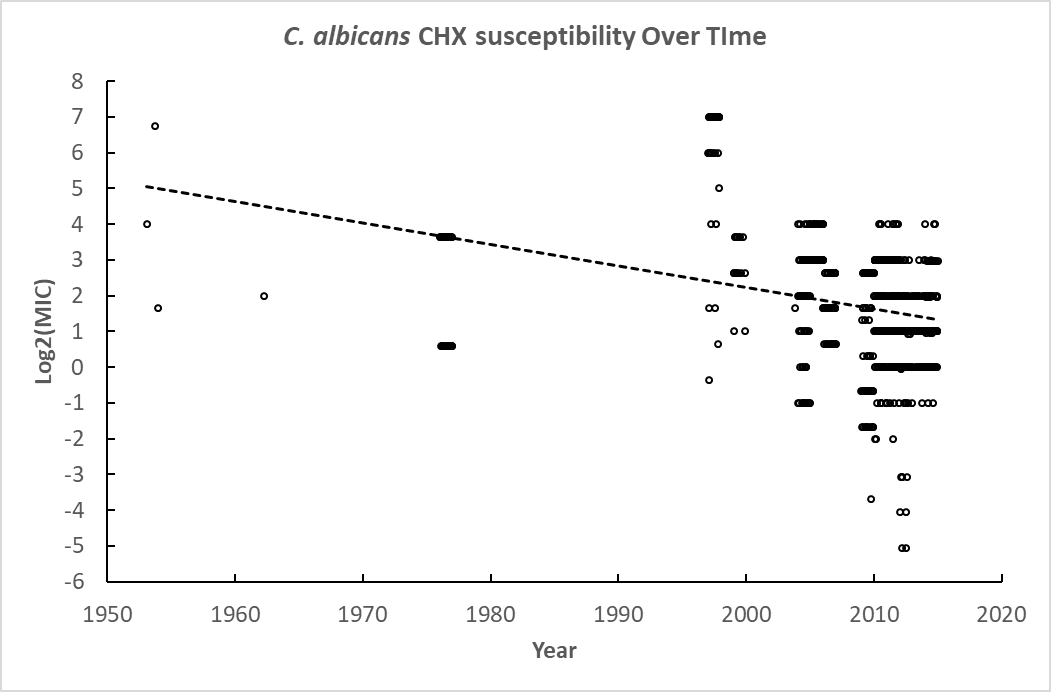


Figure S12. Linear regression analysis log_2_(MIC) values for CHX susceptibility compiled from *C. albicans* strains isolated over approximately 60 years.

The slope of the line is significantly negative, even if the data are restricted to the years since 1995 (slope = 0.0906) (not shown).


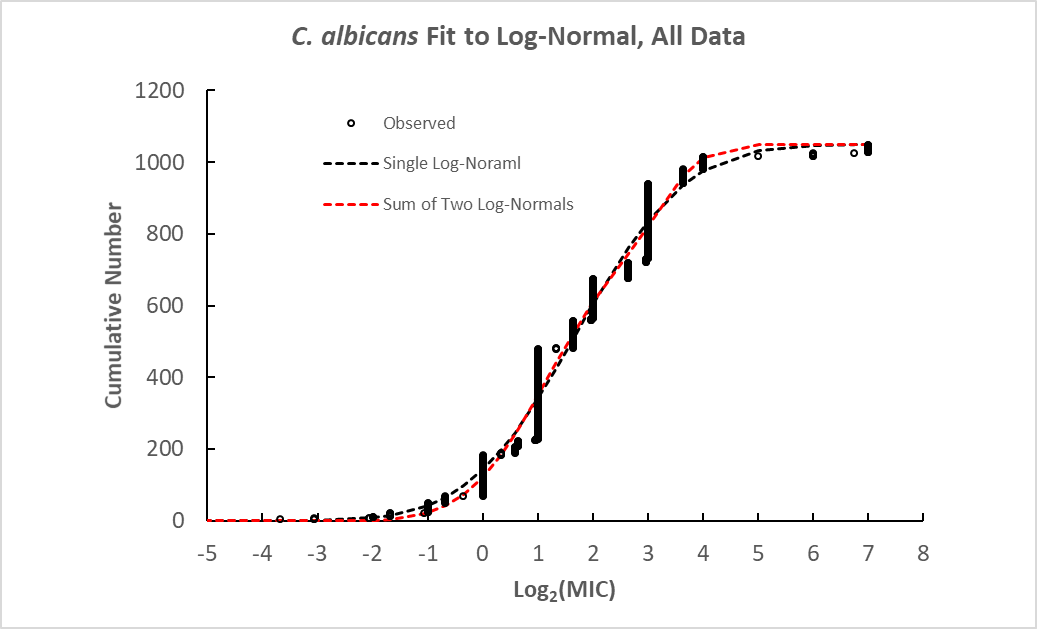


Figure S13. Analysis of C. albicans distribution of log_2_(MIC) values fitted by a single or the sum of two log-normal distributions.

| **Table S25. Log-Normal Parameters for *C. albicans*, Pooled Data**  Log_2_(MIC) values (n = 1049) | | | |
| --- | --- | --- | --- |
| **Parameter** | **Raw data** | **Fit of 1 log-norm** | **Fit of sum of 2 log-norms** |
| **Average Mean 1** | 1.75 | 1.71 | 1.19 |
| **Range Mean 1** | 1.74 to 1.76 | 1.69 to 1.72 | 1.14 to 1.22 |
| **Average Std Dev** | 1.65 | 1.55 | 1.16 |
| **Range Std Dev 1** | 1.63 to 1.67 | 1.52 to 1.56 | 1.13 to 1.18 |
|  |  | **Average N (population with lower mean)** | 252 |
|  |  | **Range N** | 237 to 269 |
|  |  | **Average Mean 2** | 3.31 |
|  |  | **Range Mean 2** | 3.26 to 3.33 |
|  |  | **Average Std Dev 2** | 0.59 |
|  |  | **Range Std Dev 2** | 0.58 to 0.60 |
|  |  | **AIC diff range** | 50 to 70 |
|  |  | **F prob range (p-value)** | 5 x 10^-12^ to 4 x 10^-16^ |

Range of parameters based on 10 permutation runs.
