# Supplementary material for: Has resistance to chlorhexidine increased among clinically-relevant bacteria? A systematic review of time course and subpopulation data
Source: PLoS One. 2021 Aug 19;16(8):e0256336. doi: 10.1371/journal.pone.0256336 (PMC8376095; doi:10.1371/journal.pone.0256336)
Supplement: Attachment — Submitted filename: Review.pdf [file pone.0256336.s005.pdf]

Has resistance to chlorhexidine increased among clinically-relevant bacteria? A systematic review of time-course and subpopulation data

Comments for Manuscript Number PONE-D-20-37954

A meta-analysis study that looks at time-course development of resistance/changes in susceptibility to CHX by looking at reported MIC values in the literature for nosocomial microorganisms. Also, the author attempted to address the degree of susceptibility to the association with antibiotic resistance.

A very comprehensive study was conducted by the author and a very large subset of data gathered from published studies was analysed, using various statistical approaches.

There are increasing reports on antibiotic resistance / multi-drug resistance, however, there is still little work done on disinfectant and or biocide resistance. Nor is there much reports on correlating antibiotic and biocide resistance. The mechanisms of biocide resistance remain unclear, although reports state that the mechanisms are similar to antibiotic resistance. The current paper “zooms” out and gathers numerous data on CHX resistance, and draws a time-line indicating how the MIC of CHX is increasing over time. A very relevant and necessary study, and novel indeed. I do agree with the authors on a national registry for MIC values, this will allow the monitoring of microbial resistance development.

However, the review in itself was difficult to assess, as firstly the paper is rather lengthy, and it could be shortened, and there is a disconnect between the methodology and the results.

Line 70-72, gives a timeline for measurement of CHX susceptibility and resistance for the particular study, dating back 20-50 years, however, literature searches included in the final analysis (line 228-246) are between 2013-2020, from which the data presented in this paper is compiled. This is misleading, the author should clearly state (which was later referred to), that the isolates date back in some of the studies 20-50 years, date of isolation. If there is literature stating that exposure to CHX dates back 20-50 years than this should be referred to / or the strains are all CHX susceptible. If so, this should be a search parameter in the data filtering.

The dates for the research papers assessed/included and the dates for the reporting of results also do not correlate. I might have missed this (maybe it has been reported on in the supplementary material), but this brings in an element of confusion. Line 270 reports on MIC values reported over an 80 year time interval, also depicted in Figure 2, but the methodology does not indicate the inclusion of sources for these earlier timelines, as filtering was applied to the literature searches to eventually only include papers that met the parameters set.

Gathered from the data, and I could be wrong, as I am not a statistician, it seems different statistical parameters were applied to the different subsets of data representing the different microbial populations. Seemingly creating statistical bias.

Line 197-203; (in short), the number of permutation runs, and comparison of parameter values from the number of runs, as an estimator of result stability. The number of permutation runs was 80, according to this section. However, for some microbial organisms, more permutation runs were performed, see Table 1; 100 permutations runs were performed for *P. aeruginosa.* It appears that the introduction of variability was not kept to a minimum.

Although mentioned, there is some disconnect between the methodology, results and discussion, regarding CHX and colistin, this is, however, extensively discussed in the discussion. However, the use of colisitin coinsiding with CHX use was not listed as an inclusion criterion, and the author does not mention why this particular antibiotic is of interest.

Editorial suggestions:

Line 228-246, can be combined and this section can be shortened.

How data is presented in figures can be improved, especially in figure 19, where MSSA and MRSA S. aureus are indicated (the author did use colours), but a better presentation would be different shapes (e.g. triangles and circles). This goes for the majority of the graphs, the presentation makes it difficult to follow.

The extensive information given in the section from line 252-266, should be kept to the relevant microbial organisms discussed in the paper. Line 264 seems misleading as the author refers to data compilation extending over 20 years, but based on the inclusion and exclusion criteria for the literature searches, the published data used does not date that far back.

The author made a great effort to discuss the relationship between CHX and antibiotic resistance, in this case, colistin. The discussion can be shortened, and some general information should be given, e.g. why the relationship of CHX and colistin was looked at, etc. This type of information might not necessarily be self-evident.

Ps. aeruginosa vs P. aeruginosa?, I am not aware of the use of Ps.aeruginosa, if this is correct then I accept.
